# Supplementary material for: Novel Driver Strength Index highlights important cancer genes in TCGA PanCanAtlas patients
Source: PeerJ. 2022 Aug 11;10:e13860. doi: 10.7717/peerj.13860 (PMC9375969; doi:10.7717/peerj.13860)
Supplement: Supplemental Information 2 [file peerj-10-13860-s002.zip › COHORTS/genes plots/detailed/2021_8_16_14_9_distribution_events_detailed_33.pdf]

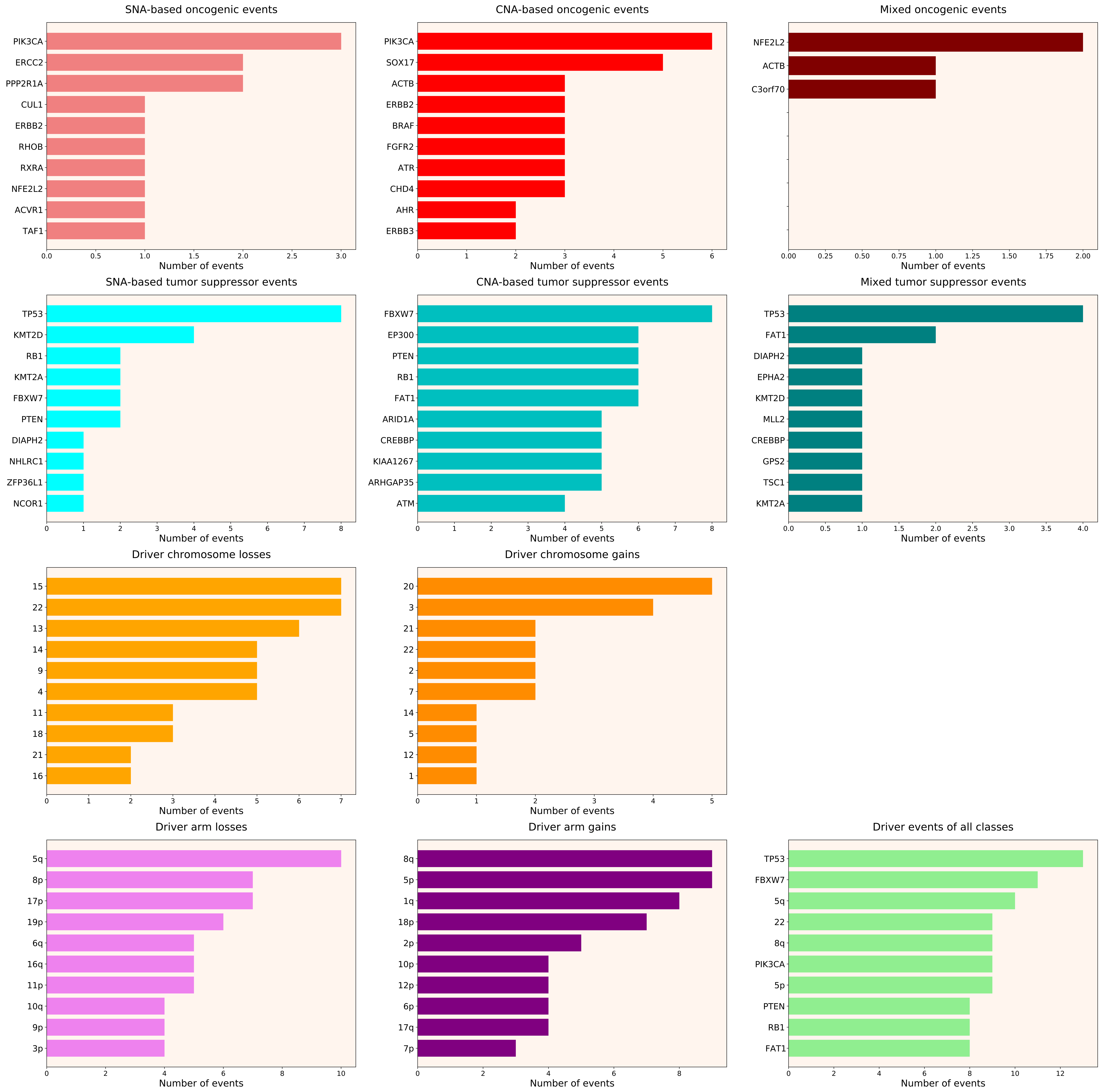
SNA-based oncogenic events
PIK3CA
ERCC2
PPP2R1A
CUL1
ERBB2
RHOB
RXRA
NFE2L2
ACVR1
TAF1
Number of events
CNA-based oncogenic events
PIK3CA
SOX17
ACTB
ERBB2
BRAF
FGFR2
ATR
CHD4
AHR
ERBB3
Number of events
Mixed oncogenic events
NFE2L2
ACTB
C3orf70
Number of events
SNA-based tumor suppressor events
TP53
KMT2D
RB1
KMT2A
FBXW7
PTEN
DIAPH2
NHLRC1
ZFP36L1
NCOR1
Number of events
CNA-based tumor suppressor events
FBXW7
EP300
PTEN
RB1
FAT1
ARID1A
CREBBP
KIAA1267
ARHGAP35
ATM
Number of events
Mixed tumor suppressor events
TP53
FAT1
DIAPH2
EPHA2
KMT2D
MLL2
CREBBP
GPS2
TSC1
KMT2A
Number of events
Driver chromosome losses
15
22
13
14
9
4
11
18
21
16
Number of events
Driver chromosome gains
20
3
21
22
2
7
14
5
12
1
Number of events
Driver arm losses
5q
8p
17p
19p
6q
16q
11p
10q
9p
3p
Number of events
Driver arm gains
8q
5p
1q
18p
2p
10p
12p
6p
17q
7p
Number of events
Driver events of all classes
TP53
FBXW7
5q
22
8q
PIK3CA
5p
PTEN
RB1
FAT1
Number of events
